# Lifecourse research in cancer: context, challenges, and opportunities when exploring exposures in early life and cancer risk in adulthood

**DOI:** 10.12688/healthopenres.13748.3

**Published:** 2025-03-14

**Authors:** Jennifer L. Baker, Vanessa L.Z Gordon-Dseagu, Trudy Voortman, Doris Chan, Zdenko Herceg, Sian Robinson, Teresa Norat, Helen Croker, Ken Ong, Ellen Kampman

**Affiliations:** 1 Center for Clinical Research and Prevention, Copenhagen University Hospital-Bispebjerg and Frederiksberg, University of Copenhagen, Frederiksberg, Denmark; 2 World Cancer Research Fund International, London, England, UK; 3 Department of Epidemiology, Erasmus MC, Erasmus, Rotterdam, The Netherlands; 4 Department of Epidemiology and Biostatistics, School of Public Health, Imperial College London, London, England, UK; 5 International Agency for Research on Cancer (IARC), World Health Organisation, Lyon, France; 6 AGE Research Group, Newcastle University, Newcastle upon Tyne, England, UK; 7 MRC Epidemiology Unit, Institute of Metabolic Science, University of Cambridge, Cambridge, England, UK; 8 Division of Human Nutrition and Health, Wageningen University & Research, Wageningen, The Netherlands

**Keywords:** Lifecourse, Cancer, Risk factors

## Abstract

As the global population ages, and rates of modifiable risk factors for cancer change, cancer incidence and mortality continue to increase. While we understand many modifiable risk factors related to diet, nutrition, bodyweight, and physical activity in adulthood that influence cancer risk, how exposure during childhood, adolescence, and young adulthood impacts cancer risk is less clear. This is partly because the timeline from initial mutation to cancer development and diagnosis can span several decades. This long latency period creates methodological, ethical, and financial issues; as well as resource and feasibility challenges in the design, implementation, and data analysis of lifecourse studies. As such, the large majority of lifecourse studies are observational, often using recall data which has inherent bias issues. Concurrently, a new research era has begun, with mature birth cohort studies that are phenotyped/genotyped and can support studies on adult cancer risk. Several studies and consortia contain information spanning the lifecourse. These resources can support association, mechanistic and epigenetic investigations into the influences of multi-disciplinary (e.g. genetic, behavioural, environmental) factors, across the lifecourse and critical time periods. Ultimately, we will be able to produce high-quality evidence and identify how/when early life risk factors impact cancer development and survival.

## Plain language summary

Cases of cancer are increasing across the world as the population ages and how many of us do things that affect our risk of getting cancer changes. Many of the things that change are cancer risk have been explored in studies of adults - rather than children. The result is that we know very little about how our behaviour in younger years might change our cancer risk as we age. One of the reasons for this is that cancer is a complex disease with many factors contributing to its development. It can also take several decades for this disease to develop - for studies to properly understand this they would have to follow people across these decades and that can often be impossible due to financial and time limitations.

Recently, new research has begun to look at ways of understanding how behaviour during childhood can change our cancer risk. Studies have now begun to use data from several studies at once, or look at our genes for answers. These studies enable us to look at the biological aspects of behaviour and cancer as we age, as well as the impact of environment upon our cancer risk. This research enables us to better understand how and when early life might be impacting cancer developing and whether or not individuals survive the disease.

## Introduction

Globally, approximately 20 million individuals were diagnosed with cancer in 2022 ^1^. For incidence, the three leading cancers for women are breast, lung, and colorectum while for men they are lung, prostate, and colorectum ^1^. Cases of cancer are predicted to increase to approximately 32.6 million by 2045, representing a 63.4% increase from 2022 numbers ^1^. It is important to note that this figure is derived from demographic extrapolations and does not take into account time trends in incidence nor in rates of risk factor exposure. A second report also estimates that, by 2070, incidence will have doubled ^2^. The increase is likely to be felt most keenly in developing, rather than developed, countries due to demographic changes, as well as changing rates of cancer-related risk factors (modifiable and non-modifiable), such as the transition from communicable to non-communicable diseases as well as underweight to overweight and obesity ^3^.

There are hundreds of different types of cancer, and each has its own unique characteristics. Nonetheless, all cancer results from genetic changes that cause the abnormal functioning of cells causing them to undergo uncontrolled growth ^4^. The timeline from the first mutation to the clinical detection of cancer is typically a long multi-stage process that can span decades. Thus, when examining which factors may affect an individual’s risk of cancer, using a lifecourse research approach is very relevant as it examines biological, environmental, behavioural, and social factors, alone or in combination, across life and even generations ^5, 6^.

Systematic literature reviews facilitated and funded by organisations such as World Cancer Research Fund/American Institute for Cancer Research (WCRF/AICR) and undertaken by universities and institutions worldwide demonstrate consistent associations between various factors related to diet, nutrition, body size and physical activity and increased risk of several site-specific cancers ^7, 8^. However, the epidemiological evidence, established mostly within European and North American studies, has focused mainly on exposures during adulthood. As such, more information is needed about effects of early life exposures and exposures to modifiable risk factors across the lifecourse from diverse populations to improve cancer prevention programmes. In this paper we discuss the opportunities and challenges of using a lifecourse approach in research on cancer risk.

## Lifecourse exposures and cancer

In the context of cancer research, the lifecourse framework theorizes that cancer risk factors can exert effects through exposures at critical periods, through an accumulation of exposures over time, or both ways ^9, 10^. A well-known example of a risk factor exposure at a critical period is that of women who took diethylstilbestrol (DES) during their first trimester of pregnancy. This medication altered the development of their daughters during foetal life and resulted in them having high risks of developing vaginal and cervical clear cell adenocarcinoma as adults ^11^. The premise that the accumulation of exposures across the lifecourse affect cancer risk can be illustrated by sunburns; as the total number of lifetime sunburns increases, so does the risk of melanoma ^12^. Tobacco use is an example of a combination of critical periods and cumulative exposure in relation to cancer risk. Further, levels or exposure to modifiable risk factors may change over time as behaviour changes. Current research tends to use a single measure of exposure, further research is needed to better understand how ongoing exposure or at multiple time points across the lifecourse impacts cancer risk. For factors such as sun exposure, it is likely that multiple exposures across early life are needed to increase cancer risk. For other factors, such as obesity, it may be that the ongoing systemic perturbations caused by them contribute to increased cancer risk.

Adolescence and young adulthood are important periods for acquiring smoking behaviours ^13^ with more than 85% of adult smokers starting before the age of 18 years ^13^. Investigations of smoking trajectories demonstrate that smoking often starts in adolescence, progresses through young adulthood, and becomes an entrenched behaviour by the age of 25, which is then carried on into later life ^14^. Adolescence may be a critical period when there is an enhanced biological vulnerability of the developing lungs to effects of cigarette smoking in relation to smoking-related morbidity than at older ages ^15^, but effects on cancer are not yet known. Further, reflecting the subsequent higher cumulative exposure (a function of intensity and duration), individuals who begin smoking earlier in life have a higher risk of smoking-related morbidity and mortality, including for lung cancer, compared with those who began later ^16– 19^. Whether the biological effect (in terms of how a behaviour or exposure impacts health) is the same for each lifecourse phase or changes with age is yet to be clearly understood.

## Prenatal and early life exposures

Periods of rapid growth from prenatal life to adolescence may represent periods of vulnerability for the later risk of cancer, a concept which fits into the Developmental Origins of Health and Disease (DOHaD) hypothesis ^17, 19^. Despite the plausibility of exposures during early life being related to cancer risk, this field of research is still not as well-established as links between early life exposures and, for example, cardiovascular disease. In part, this is because there is a lack of intermediate endpoints for investigations of cancer. Numerous studies have, however, shown that birth weight and length, as indicators of the foetal environment and inheritance, are linked to cancer risk ^20^. Reflecting the diversity of cancer aetiologies ^21^, the associations vary by cancer site and type. Although birth weight and length are not suitable direct targets for cancer prevention efforts, their associations with cancer yield insights into the potential early origins of cancer risk that can be explored further in mechanistic studies.

Higher body mass index (BMI: kg/m ^2^) ^22^ and body fatness during childhood, assessed by self-report via pictograms ^23^, are associated with higher risks of many, but not all, cancer types that are also associated with overweight and/or obesity in adulthood ^24^. Further, having a trajectory of a high BMI across childhood is positively associated with the risk of adult obesity-related cancers in men and women and, conversely, with a lower risk of pre- and post-menopausal breast cancer in women ^25^. Evidence from Mendelian Randomisation studies further support the inverse association between childhood BMI and breast cancer ^26^. Although these findings are intriguing, these studies either did not account for exposures to other relevant factors across the lifecourse (e.g. smoking, nutrition, physical activity, adult obesity) ^22^ and/or relied upon information about childhood recalled decades later by the study participant (e.g. past body size and shape recalled via a pictogram or diet recalled via a survey) ^23^. How caloric restriction, either long-term or during a clearly defined period of time such as during the Dutch famine (Hunger Winter), impacts later cancer risk (either overall or for site-specific cancers) is an under-investigated area of research.

Much remains unknown about potential biological mechanisms linking body size at childhood ages to cancer risk and the additional influence of other moderating or mediating exposures across the rest of the lifecourse. Further, whether there are critical periods during early life when remission from having a high BMI or body fatness may reduce the later risk of cancer remains largely unknown. Although a few studies suggest this may occur ^27, 28^, details beyond body size, weight, and shape are lacking, making the picture incomplete and warranting further investigation.

A growing body of evidence reports childhood height and linear growth to be positively associated with many forms of cancer – although the limitations mentioned previously (e.g., lack of information about confounders) are often present ^20, 22^. A 2020 review of studies using the Copenhagen School Health Records Register found child height was positively associated with several cancers including, but not limited to, those of the breast, colon, endometrium, kidney, oesophagus, ovaries, and prostate ^22^. Further, above average linear growth was associated with an increased risk of cancers of the breast, colon, kidney, and prostate. These associations reflect the positive associations observed between attained adult height and cancer risk ^20^. Menarche, which is at the end of a girl’s rapid growth during puberty, has also been investigated in relation to the later risk of cancer. While the evidence is currently limited, younger age at menarche appears to be associated with higher risks of several forms of hormone-related cancers in mid-life ^29^. Neither height nor age at menarche are directly modifiable, thus limiting their immediate applicability to cancer prevention, but they highlight the potential importance of exposures at ages when growth occurs and expand opportunities for further mechanistic studies.

Another area that requires further development of the evidence-base relates to dietary habits and physical activity at early ages, and even during pregnancy, and cancer risk during childhood and adulthood. A small body of evidence suggests that a diet high in fruit, vegetables, and fibre, and low in red meat during childhood and adolescence may be protective in terms of adult breast and colorectal cancer ^30, 31^. Evidence, however, is even more sparse for other cancer sites ^30, 31^. Exposure to severe energy restriction during early life is positively associated with breast cancer in women and prostate cancer in men, but the mechanisms remain speculative ^32^. For childhood physical activity, again, there is a paucity of evidence; much remains to be learned about other modifiable cancer-related risk factors ^30^. As with smoking, diet and physical activity behaviours often track into adulthood and warrant further investigation ^33^.

Commentaries have reported a need for more research focused upon exposure to modifiable risk factors during prenatal life and future cancer risk ^34^. For example, how the diet of the mother, and therefore the nutrients available to the foetus, might function as a risk factor for future cancer. The same is true for risk factors such as alcohol consumption and smoking during the prenatal phase – there is a limited but growing body of evidence linking smoking by the mother and father with increased risk of some site-specific cancers in early life ^35, 36^. Although yet to be fully understood, mechanistic pathways leading from prenatal risk factors to cancer may be via alterations to adiposity, as well as age at menarche and hormonal factors, in the offspring via placental or epigenetic alterations which then impact cancer risk ^37– 39^.

Taken together, these studies demonstrate that prenatal and childhood anthropometry, and possibly childhood diet and physical activity, may indicate altered risks for several site-specific cancers in adulthood. Whether the links are causal or independent of later life exposures remains to be fully determined. Nonetheless, findings from previous studies highlight the relevance of early life for mechanistic studies as well as the potential for prevention.

## Potential mechanisms linking early life exposures to cancer risk

There are a wide variety of mechanisms plausibly linking early life exposures to later cancer risks – with the mechanisms differing depending on the site-specific and type of cancer under investigation. These include core cellular processes, including epigenetic regulation and DNA damage response pathways, systemic processes including inflammation to alterations in hormone levels and secretion, alterations in childhood linear growth, body composition and adipose tissue distribution, as well as the early establishment of lifestyle behavioural patterns and preferences.

### Cellular processes

Epigenetics is proposed as a key mechanism in shifting the gene expression programme in response to environmental exposures and endogenous cues ^40– 42^. For example, consistent epigenetic differences have been found in infants from Gambia, a predominantly agricultural society, depending on which season they were conceived in, and thus exposed to large differences in maternal nutrient status, diet composition, and energy balance ^43– 45^. The epigenome acts as a ‘sensor’ of an exposure and can be a ‘mediator’ of health and disease outcomes; thereby linking early life exposures to later cancer ^41, 42^. Epigenetic reprogramming plays a critical role in embryonic development, the establishment and maintenance of gene expression programmes in stem/progenitor cells, and cell lineage commitment ^46^. Therefore, unscheduled changes in the epigenome pattern during this window of vulnerability may be propagated along with other changes in gene expression programmes leading to cancer ^47– 49^. Supporting this, a study nested in a cohort revealed “epigenetic loci” associated with risk of developing several types of cancer in later life ^50^. Nonetheless, further studies are needed to substantiate a contribution of epigenetic mechanisms to cancer in later life.

Other cellular processes potentially linking early life exposures to cancer include DNA damage sensing and response mechanisms. These mechanisms have also been implicated in cell replication and the early life establishment of the ovarian oocyte pool size, thus making them relevant to ovarian cancer ^51^. Further, haematopoietic clonal histories reconstructed from whole-genome sequencing of circulating white blood cells has shown that the acquisition of somatic driver mutations in DNA may occur very early in life-already during foetal life and early childhood- many decades before the manifestation of myeloproliferative disease ^52^. These findings suggest that there is much to be learnt about the potential mechanisms linking early life development to later cancer risks.

### Early life growth

Childhood growth comprises three distinct and overlapping phases of infancy, childhood, and puberty. These have different biological drivers and may respond differentially to early life exposures ^53^. The infancy growth phase is particularly susceptible to changes in nutrition. The main hormonal regulators of the childhood phase until puberty are growth hormone (GH), insulin-like growth factor 1 (IGF-1) and thyroid hormones ^54^. During the pubertal phase, sex steroid hormones are the main biological drivers ^54^.

During these phases, there are profound changes in not only linear growth but also body composition. Normal growth is accompanied by increases in both fat free mass and fat mass. An excessive accumulation of fat mass and/or the central distribution of fat mass, however is linked with insulin resistance ^55^, chronic inflammation ^56^, and alterations in hormone secretion and sensitivity ^57^; each of which may link to the later risk for cancer. Growth patterns characterised by a combination of lower birth weight and rapid postnatal are associated with subsequent higher adrenal androgen secretion and earlier puberty timing, which in turn is linked to the later risk of some cancer types ^20^. While it is possible that this early life body size trajectory may in part reflect the effects of a common genetic susceptibility to earlier growth and physical maturation, this pattern is also associated with known non-genetic determinants of early growth, including primiparity, maternal smoking and infant formula feeding ^58^. Further, as the GH-IGF-1 axis is a key regulator of growth in height and as it implicated in carcinogenesis ^59^, this is a potential mechanism underlying the positive associations between height and many forms of cancer ^60^.

### Early life establishment of lifestyle behavioural patterns and preferences

Early life is also an important period for learning behavioural patterns and preferences that may last a lifetime. During the first years of life, children establish autonomous feeding behaviours, including food acceptance and preferences which track into adolescence and adulthood ^61^ and thereby potentially impact later risks of cancer. A child’s acceptance of novel foods (for example, previously untried fruits and vegetables) may be increased by early repeated exposure to support familiarisation as well as associative learning ^62, 63^. Lifestyle patterns relating to diet and physical activity that are identifiable in infancy show tracking during childhood and a relation to socioeconomic position ^64^. If unfavourable patterns are established early and persist, this may link with the later risk of cancer via effects at critical periods and cumulative effects of exposure to these risk factors across the lifecourse.

## Methodological challenges in studying lifecourse exposures and cancer risk

The process of cancer development in humans often spans several decades ^65^. Although randomised controlled trials (RCTs), provide gold standard evidence for cause and effect- if well-designed and well-conducted, in this field they are limited by feasibility and ethical concerns. The long latency creates methodological, ethical, and financial resources challenges in the design, implementation, and data analysis of lifecourse studies. As such, the large majority of lifecourse studies are observational.

Particularly relevant to lifecourse cancer research is the issue of bias. A key concern is that most observational studies on cancer risk factors focus on adult ages and are based upon cohorts recruited in mid-life. There is an inherent risk of survival bias (a form of selection bias) as these studies can only ever include those who survive to the age at which the information is collected; this may have an impact on study results, depending upon the rate of survival to adulthood within the study setting. Additionally, in these types of studies, information on early life factors is typically obtained through self-report and recall, which may introduce imprecision in the information and introduce bias if measurement errors are related to the outcomes. Further, bias from residual and unmeasured confounding cannot be ruled out, even when exposures are meticulously measured, as it is impossible to measure every potential confounder and mediator that occurs over the span of a lifecourse study.

Another observational design that is highly relevant for lifecourse research is birth-cohort studies. These provide the best temporality evidence for drawing causal inference about the early life exposure–adult cancer relationships, because they either not affected by or less impacted by the above-mentioned types of biases. To date, however, they have been too small to support studies on cancer, have too short of a follow up period for cancer cases to develop, and many have suffered from substantial loss to follow-up, and invalid, unreliable and/or incomplete longitudinal exposure and covariate data collection. Historical cohort studies that utilise existing medical or registry records or specimens are useful, but these can be limited by the availability of information and time periods of the exposure and covariate data collection, hindering the examination of potential interactions between early and later life exposures or accumulated risk.

Other studies designs are relevant to lifecourse cancer research. Case-control studies are efficient for studying rare types of cancer. Their limitations relate to being able to determine the temporal relationships needed in lifecourse studies. Case-control studies often suffer from information bias as the outcome has already occurred, while exposure may have taken place years prior. This increases the risk of recall bias/over-reporting of the exposure among cases. Mendelian Randomisation studies are increasingly being used in lifecourse cancer research. These studies exploit the principle of randomly allocated genetic variants as instruments for the exposures of interest and are theoretically not influenced by confounding and reverse causation. These studies are useful for strengthening the findings from other studies if the key assumptions are met ^66, 67^. Nonetheless, how data resources were generated, and issues of bias still require consideration in the interpretation of results.

Apart from data availability, the modelling and statistical analysis of exposures across the lifecourse presents a series of challenges. There is not a ‘one size fits all approach’, and the model used depends on the question being asked. Conceptual frameworks and directed acyclic graphs are commonly used to illustrate the relationships among the factors under study and for guiding how the statistical models should be set up in epidemiological studies ^68^. Due to the time element, different analytic approaches are needed to handle repeated observations of the exposures and potential confounding and mediating variables. Further, different methods are needed to handle the correlations among these factors over time as well as potential interactions. For example, many exposures such as BMI or smoking behaviour, are correlated with each other (or show ‘tracking’) across the lifecourse. Disentangling the relative contributions of repeated exposures to the risk of cancer is not necessarily as simple as including all measures at the same time in one model. Additionally, analyses of longitudinal data are often challenged by missing data and sample size attrition. The use of machine learning is also on the horizon in cancer lifecourse research, with an algorithmic modelling approach to predict or discover patterns in the data ^69^. Methodological advances are being made in lifecourse analyses, but much remains to be done ^70^.

## Opportunities and future perspective

We will soon enter an era where deeply phenotyped and genotyped birth cohort studies are old enough to support studies on adult cancer risk. This offers the opportunity to undertake research able to utilise follow-up periods that are long enough to explore the effectively explore the associations between early life exposures and cancer in later life. Further, through international consortia, numerous cohort studies with information on birth, childhood, adolescence, early- and middle-adulthood exposures are similarly available to support these kinds of studies ( Table 1). These resources can support both association and mechanistic investigations into the independent and synergistic influences of the multi-disciplinary (e.g., genetic, biological, behavioural, environmental, socioeconomic) risk factors, across early and later phases of life and/or across specific critical time periods, on site-specific cancer development. Finally, it is often the case that individual studies lack the necessary number of cases to explore early life exposure and cancer risk. The use of consortia data or linked data from individual studies has the potential to overcome this issue.

**Table 1.** Examples of data resources (including their websites) that can potentially support lifecourse research into cancer risk.

Name and website	Aim and contents
Birth Cohorts https://birthcohorts.net	“To facilitate the exchange of knowledge and collaboration between cohorts and researchers. In addition, we would like to provide administrators, policy makers and other stakeholders with information about available cohort data on health and its determinants.” ^77^
Cancer Epidemiology Descriptive Cohort Database (CEDCD) https://cedcd.nci.nih.gov/	The data base “contains descriptive information about cohort studies that follow groups of persons over time for cancer incidence, mortality, and other health outcomes. The CEDCD is a searchable database that contains general study information (e.g., eligibility criteria and size), the type of data collected at baseline, cancer sites, number of participants diagnosed with cancer, and biospecimen information. All data included in this database are aggregated for each cohort; there are no individual level data. The goal of the CEDCD is to facilitate collaboration and highlight the opportunities for research within existing cohort studies.” ^78^
EU Child Cohort Network https://lifecycle-project.eu/	In addition to research, it “will set up a European pregnancy and childhood cohort, the EU Child Cohort Network, which brings together extensive existing data from more than 250,000 European children and their parents” ^79^
International Childhood Cancer Cohort Consortium (I4C) https://www.mcri.edu.au/research/projects/ international-childhood-cancer-cohort- consortium-i4c	“We aim to examine associations between environmental exposures and the incidence of childhood cancers by pooling prospective population data from one million pregnant mothers and their babies.” ^80^

Future studies examining epigenetic mechanisms and prenatal programming will contribute substantially to what is known about links between early life exposures and cancer susceptibility in later life. Epigenetic hotspots may serve as powerful targets for investigating epigenetic mechanisms underlying the impact of early life exposures on cancer. The emergence of integrative systems biology models, including the epigenome and other omics layers as well as functional pathways annotation, toxico-genomics data, and phenotype data should facilitate these investigations ^41^. Once validated, molecular signatures associated with early life exposure factors could potentially serve as objective markers for predicting cancer ^71, 72^.

Despite progress in lifecourse studies on cancer in high-income countries, studies from middle- and low-income countries are sparse ^73, 74^. In large part, this is due to the expense and infrastructure requirements for establishing and maintaining these types of data resources and related healthcare systems ^74^. Given that a large percentage of the world’s population lives in low- and middle-income countries where cancer-related risk factors are different, and that rates of cancer mortality are high in these areas ^75^, this critical gap in knowledge needs to be addressed. Existing cohort consortia ^76^ and emerging large-scale projects ( https://h3africa.org/) may provide such research opportunities.

The work of the WCRF/AICR within the Global Cancer Update Programme (CUP Global) reviews the large body of epidemiological evidence related to diet, nutrition, body fatness and physical activity and cancer risk. Through systematic reviews and meta-analyses, we can identify associations between exposure and cancer outcomes. We have also begun to explore the biological mechanisms that underpin these associations via a collaboration with IARC. Ultimately, through examining exposures across the lifecourse, the goal of WCRF/AICR in CUP Global is to generate high quality evidence that identifies how and when cancer risk factors act across the lifecourse so that cancer can be prevented, and survival improved.

## Disclaimer

Where authors are identified as personnel of the International Agency for Research on Cancer/ World Health Organization, the authors alone are responsible for the views expressed in this article and they do not necessarily represent the decisions, policy or views of the International Agency for Research on Cancer/ World Health Organization.

## Ethics and consent

Ethical approval and consent were not required.

## Data Availability

No data are associated with this article.
