# Supplementary material for: Lifecourse research in cancer: context, challenges, and opportunities when exploring exposures in early life and cancer risk in adulthood
Source: Health Open Res. 2025 Mar 14;6:16. Originally published 2024 Jul 1. [Version 3] doi: 10.12688/healthopenres.13748.3 (PMC11836561; doi:10.12688/healthopenres.13748.3)
Supplement: Peer review history

# Reviewer report: Reviewer response for version 3

**DOI:** 10.21956/healthopenres.14946.r29310

**Published:** 2025-03-18

**Authors:** Savio George Barreto

**Affiliations:** 1 Surgery, College of Medicine and Public Health, Flinders University, Adelaide, South Australia, Australia

Some of the concerns raised have been addressed.

Is the topic of the opinion article discussed accurately in the context of the current literature?

Partly

Are arguments sufficiently supported by evidence from the published literature?

Yes

Are all factual statements correct and adequately supported by citations?

Partly

Are the conclusions drawn balanced and justified on the basis of the presented arguments?

Partly

Reviewer Expertise:

Young-onset cancer; early-onset cancer

I confirm that I have read this submission and believe that I have an appropriate level of expertise to confirm that it is of an acceptable scientific standard.

# Reviewer report: Reviewer response for version 2

**DOI:** 10.21956/healthopenres.14912.r29275

**Published:** 2025-02-18

**Authors:** Esther de Vries

**Affiliations:** 1 Department of Clinical Epidemiology and Biostatistics, Pontificia Universidad Javeriana, Bogotá, Colombia

This is a very clearly written manuscript on a methodological issue of much importance in cancer research and potential growth in the coming years. It gives a nice, concise overview of the advantages and difficulties.

 I have no major comments, just some minor suggestions to improve the contents or readability.

 a) In the view of the life-course, which is central to this manuscript, it would be very interesting to cite, as counterpart to effects of overweight, in the BMI paragraph the contributions of the "Hunger Winter" study which clearly demonstrated effects of a very defined, relatively short period of caloric restriction at different moments of life on future cancer risk. This is now limited to reference 32 and breast cancer in the text.

 b) In the section on "methodological challenges", in this sentence I would suggest to include the word "typically" or "usually": "Additionally, in these types of studies, information on early life factors is typically obtained through self-report and recall, " - as sometimes there is a precise measurement, for example in the case of having received certain medication or radiation or having been infected.

 c) A methodological challenge that is not explicitly mentioned is that exposures such as "tobacco smoking" or "anticonceptive use" are often used - but the exact exposure may have substantially changed over time.

 d) As well, selective participation in large population-based cohorts has been mentioned as problematic, even when exposures are meticulously measures - because of unmeasured confounding factors.

**Some small corrections:**

 e) The second time reference number 1 (referring to Cancer Today) is mentioned, they refer to the predictions for 2045 - and the correct citation that second time would be to Cancer Tomorrow (https://gco.iarc.fr/tomorrow/en). In this sentence referring to 2045 it is useful to mention that this is based purely on demographic extrapolations, without taking into account time trends in incidence nor in risk factor exposure prevalence.

 f) In that same sense, the last sentence of the first paragraph refers to prevalence of exposure to cancer-related modifiable risk factors rather than rates of risk factors.

Is the topic of the opinion article discussed accurately in the context of the current literature?

Yes

Are arguments sufficiently supported by evidence from the published literature?

Yes

Are all factual statements correct and adequately supported by citations?

Yes

Are the conclusions drawn balanced and justified on the basis of the presented arguments?

Yes

Reviewer Expertise:

Cancer Epidemiology and cancer surveillance

I confirm that I have read this submission and believe that I have an appropriate level of expertise to confirm that it is of an acceptable scientific standard.

# Response

**Published:** 2025-03-05

**Authors:** Vanessa Gordon-Dseagu

**Affiliations:** World Cancer Research Fund International, London, England, UK

We thank the reviewer for their time and responses to our manuscript. Below we offer our comments and amendments in response to their review.  

1. We agree with the reviewer and have added a sentence to the paragraph about BMI.
2. The word ‘typically’ has been added.
3. A sentence has been added to the section about tobacco to capture this point.
4. The last sentence of the 2 ^nd^ paragraph in the ‘Methodological challenges’ section has been amended to make this point clearer.

**Small corrections:**

1. This reference has been updated and an explanatory sentence added to the introductory paragraph.
2. This has been amended to include both modifiable and non-modifiable risk factors.

# Reviewer report: Reviewer response for version 2

**DOI:** 10.21956/healthopenres.14912.r29273

**Published:** 2025-02-04

**Authors:** Savio George Barreto

**Affiliations:** 1 Surgery, College of Medicine and Public Health, Flinders University, Adelaide, South Australia, Australia

I am grateful for the opportunity to review this interesting manuscript given my personal interest in early-onset adult cancers over two decades both treating young adults living with cancer as well as researching the topic. There have been a few publications over the years repeatedly stating the usual aetiological factors associated with cancer causation. It appears that we are missing the point in terms of causation of early-onset adult cancers. How do we explain the role of these ‘standard’ aetiological factors in causation when the incidence of most cancers in people above 50 years (even those cancers without screening programmes) have either remained steady, or are on the decline despite these older individuals having been exposed to the putative risk factors much longer than young people? The answer lies in two critical factors:

1. The timing of the exposure in terms of an individual’s life course, and
2. The ability of that exposure to trigger a stress response in that individual sufficient enough to lead to epigenetic modifications. This differential effect of the same noxious stimulus on different individuals is the result of not only the dose of exposure but the individual’s adaptive responses (neurohormonal and immune), and maybe even their microbiome.

The authors of this paper have rightly considered these factors and elaborated on them through this manuscript. The early life period remains a critical period in an individual’s life when exposures to noxious stimuli (unprotected sun exposure, drugs, alcohol, smoking, unhealthy high calorie foods) may be the result of poor choices from a lack of education, peer pressure, or driven by factors such as socio-economic status. An equally concerning period is the perinatal period where maternal and / or paternal choices in terms of use of drugs, alcohol, smoking, unhealthy high calorie foods at the time of conception of the baby and during pregnancy could ‘stress’ the *in utero *foetus. This, too, has been beautifully discussed by the authors.

 A concern I have with this manuscript is with the decision of the authors to focus solely on a significant exposure at a single time period as proposed by the DOHAD hypothesis. Cancer is not the same as a chronic disease. I agree that it would seem intuitive to look at them with the same lens because: a) they have similar putative aetiological factors (from a DOHAD perspective), and b) the association between the two as presented by Tu et al. ^1^ – which actually signals back to the aetiological factors in point c). Chronic diseases are known to impact the neurohormonal and immune adaptive responses ^2^ and even the microbiome ^3^. However, the prevalence and incidence of chronic diseases far exceeds that of cancer (thankfully). Cancer is a genetic disease. The pioneering work of Professor Knudson ^4^ demonstrated the need for two hits. Important work by Lahouel et al. ^5^ helped clarify the timing of these two hits in sporadic cancers. Thus, taking on board all of these known facts and the evidence in the literature at the time, Professor Pandol and I proposed the PELICan (Perinatal and Early Life Influences on Cancer) hypothesis ^6^ stating that stressor events in the perinatal and early life period (childhood, adolescence or early adulthood) are essential for the causation of early-onset cancers. The key point is the need for two hits – two different time periods with the second hit necessarily occurring in the late adolescence or adult life. Thus, i request the authors to consider this in the discussion of their manuscript as well as in the data collection and analyses they propose that need to be undertaken. In my own research we have found that analyses focusing on a single period could turn up a significant risk factor. We recently demonstrated that children conceived, or born, immediately following the Great China Famine were at an increased risk of developing early-onset genitourinary cancers. However, what we noted when we analysed the follow up data was that these females had a significantly increased risk of alcohol usage in their adult life! ^7^ From a translational point of view, too, how would the identification of a single stressor in the perinatal or early life period be of clinical relevance? It is not as though every individual with an identified single stressor event can be offered screening for every cancer. Rather, the identification of these sentinel stressor events should become the focus of primary health prevention campaigns advising individuals to avoid the noxious stimuli mentioned above in adolescence through to their adult life.

 The next point is regarding consortia. The authors correctly highlight the need for consortia to truly improve patient numbers to permit the conduct of meaningful statistical analyses. Over the last 5 years I have reached out to many LifeCourse teams globally to garner their support to study early-onset cancers because I, too, believe these teams have the capacity to find the answers we are looking for. However, the main challenges I have faced in my interactions with Life Course data custodians have been:

1. The lack of sufficiently long follow ups (i.e. up to 50 years) to be able to pick up all the cancers developing in the participants’ lives. This has primarily been because these cohorts were set up to study chronic diseases or diseases in childhood and adolescence.
2. Another issue has been achieving a statistically adequate sample size of cancers to draw meaningful inferences. Most, though, not all cancers are on the rise in adults. The average incidence rates across the board vary from 0.8-1.2/100,000. This seriously precluded my ability to focus solely on early-onset adult gastrointestinal cancers. It, thus, led me to address all cancers in the first instance because of the appreciation that the risk factors are likely to be common across the cancer sites.
3. The third problem is need for all the cohorts in the consortium to have collected the same, or similar, risk factor data at specific time points in a participant’s life. To study the PELICan hypothesis, this would require perinatal, childhood / adolescent and early adulthood variables. Ever so often, the cohorts within consortia have not necessarily collected all this data.
4. Data linkage to this day remains a challenging and time-consuming process. Existing LifeCourse cohorts were set up to study chronic diseases. Thus, all of them would require data linkage to add the required variable of cancer.
5. Novel insights – the uncovering of new risk factors which are topical remain a challenge since the ability to go back in time to collect this data is often not possible. An example of this is the concern I have of the risk of early-onset adult cancers potentially in children conceived in the first year following acute significant weight loss occurring after bariatric surgery or weight-loss medications ^8^ at a time when the mother’s nutritional status is inadequate. The Great China Famine study ^7^ led to this realisation. However, after reaching out to researchers in the field, the only scientist collecting this data of offspring born to mothers post-bariatric surgery ^9^ while very supportive to work on this concept, had the oldest participant follow up at 18 years of age.
6. Lastly, International governance matters around sharing of research data. The new rules in place around data sharing and governance in the EU has certainly impacted on the ability to collaborate. I reached out to the custodians of one of the consortia listed in the authors’ manuscript. While that consortium was established to study another disease, they are now actively working with me to find cohorts. Despite proposing the study to the consortium, I am unable to serve as the Principal Investigator owing to me not being from an EU country. This has not stopped me from pursuing this important work with this consortium (where I will serve as a Consultant) since my priority remains the search for the cause of early-onset adult cancers. However, such types of governance could preclude meaningful and important collaborative research especially today when scientists are so protective of their intellectual property as is our right!  

These real-life problems plaguing the existing databases even within large consortia need to be clearly teased out in the manuscript to provide the reader with a realistic viewpoint of what lies ahead.

 In conclusion, I am pleased that these amazing scientists / authors have chosen to write on this topic. I have said it in the past and I will say it again, ““We never saw this (the problem of early-onset adult cancers) coming. But in 20 years if we don’t have databases to record this, it’s our failure. It’s negligence.” ^10^

**References**

 1.           Tu H, Wen CP, Tsai SP, et al. Cancer risk associated with chronic diseases and disease markers: prospective cohort study. BMJ 2018;360:k134.

 2.           Rubinow KB, Rubinow DR. In immune defense: redefining the role of the immune system in chronic disease. Dialogues Clin Neurosci 2017;19:19-26.

 3.           Madhogaria B, Bhowmik P, Kundu A. Correlation between human gut microbiome and diseases. Infect Med (Beijing) 2022;1:180-191.

 4.           Knudson AG, Jr. Mutation and cancer: statistical study of retinoblastoma. Proc Natl Acad Sci U S A 1971;68:820-3.

 5.           Lahouel K, Younes L, Danilova L, et al. Revisiting the tumorigenesis timeline with a data-driven generative model. Proc Natl Acad Sci U S A 2020;117:857-864.

 6.           Barreto SG, Pandol SJ. Young-Onset Carcinogenesis - The Potential Impact of Perinatal and Early Life Metabolic Influences on the Epigenome. Front Oncol 2021;11:653289.

 7.           Shuai A, Ullah S, Yu Y, et al. Perinatal Famine Exposure and Young-Onset Cancer—Lessons from China Health and Nutrition Survey. Cancers 2024;16:2537.

 8.           Barreto SG, Moy C, Pandol SJ, et al. Fecundity and Nutrient Deficiency Following Obesity Treatment: Implications for Young-Onset Cancer Risk in Offspring. Cancers (Basel) 2024;16.

 9.           Ceulemans D, Deleus E, Benhalima K, et al. Pregnancy After Metabolic Bariatric Surgery: Risks and Rewards for Mother and Child. BJOG 2024.

 10.        Ledford H. Why are so many young people getting cancer? What the data say. Nature 2024;627:258-260.

Is the topic of the opinion article discussed accurately in the context of the current literature?

Partly

Are arguments sufficiently supported by evidence from the published literature?

Yes

Are all factual statements correct and adequately supported by citations?

Partly

Are the conclusions drawn balanced and justified on the basis of the presented arguments?

Partly

Reviewer Expertise:

Young-onset cancer; early-onset cancer

I confirm that I have read this submission and believe that I have an appropriate level of expertise to confirm that it is of an acceptable scientific standard, however I have significant reservations, as outlined above.

## References

[1] Tu H, Wen CP, Tsai SP, et al. Cancer risk associated with chronic diseases and disease markers: prospective cohort study. BMJ 2018;360:k134. 10.1136/bmj.k134PMC579114629386192

[2] Rubinow KB, Rubinow DR. In immune defense: redefining the role of the immune system in chronic disease. Dialogues Clin Neurosci 2017;19:19-26. 10.31887/DCNS.2017.19.1/drubinowPMC544236028566944

[3] Madhogaria B, Bhowmik P, Kundu A. Correlation between human gut microbiome and diseases. Infect Med (Beijing) 2022;1:180-191. 10.1016/j.imj.2022.08.004PMC1069970938077626

[4] Knudson AG, Jr. Mutation and cancer: statistical study of retinoblastoma. Proc Natl Acad Sci U S A 1971;68:820-3. 10.1073/pnas.68.4.820PMC3890515279523

[5] Lahouel K, Younes L, Danilova L, et al. Revisiting the tumorigenesis timeline with a data-driven generative model. Proc Natl Acad Sci U S A 2020;117:857-864. 10.1073/pnas.1914589117PMC696952031882448

[6] Barreto SG, Pandol SJ. Young-Onset Carcinogenesis - The Potential Impact of Perinatal and Early Life Metabolic Influences on the Epigenome. Front Oncol 2021;11:653289. 10.3389/fonc.2021.653289PMC811679333996575

[7] Shuai A, Ullah S, Yu Y, et al. Perinatal Famine Exposure and Young-Onset Cancer—Lessons from China Health and Nutrition Survey. Cancers 2024;16:2537. 10.3390/cancers16142537PMC1127519739061177

[8] Barreto SG, Moy C, Pandol SJ, et al. Fecundity and Nutrient Deficiency Following Obesity Treatment: Implications for Young-Onset Cancer Risk in Offspring. Cancers (Basel) 2024;16. 10.3390/cancers16173099PMC1139424539272957

[9] Ceulemans D, Deleus E, Benhalima K, et al. Pregnancy After Metabolic Bariatric Surgery: Risks and Rewards for Mother and Child. BJOG 2024.10.1111/1471-0528.1803239663779

[10] Ledford H. Why are so many young people getting cancer? What the data say. Nature 2024;627:258-260. 10.1038/d41586-024-00720-638480960

# Response

**Published:** 2025-03-05

**Authors:** Vanessa Gordon-Dseagu

**Affiliations:** World Cancer Research Fund International, London, England, UK

We thank the reviewer for their detailed review of our paper, especially given their extensive knowledge of, and experience in, the subject matter. We too feel it is important to consider the impact of early life exposures upon future cancer risk in adulthood and look for novel ways of working (for example consortia and linked data) to better understand this complex area of investigation.  

 We agree with the point made about single/multiple/ongoing stressors and have added extra information to the manuscript – please see the end of the 1 ^st^ paragraph under the heading ‘Lifecourse exposures and cancer’.  The point on the lack of long follow-up in consortia data has been noted.

 We believe that we are entering a new era where studies will have such data available. Within the manuscript we have added this point to the 1 ^st^ paragraph under the heading ‘Opportunities and future perspective’. Within this paragraph we have also added further information related to how the use of consortia data/linked data can overcome the small number of cancer cases found within individual studies.   Finally, we thank the reviewer for their extensive description of their own experiences of undertaking lifecourse research within the context of cancer.

# Reviewer report: Reviewer response for version 1

**DOI:** 10.21956/healthopenres.14837.r28915

**Published:** 2024-11-14

**Authors:** AnnaLynn M. Williams

**Affiliations:** 1 Departments of Epidemiology and Cancer Control, St. Jude Children's Research Hospital, Tennessee, USA; 2 University of Rochester, New York, USA

The authors present a nice summary of the existing knowledge of lifecourse research in the setting of cancer etiology. In addition they provide a broad overview of the challenges that have plagued this field of research for a long time as well as several areas of opportunity on the horizon. I have only two minor comments.

 1. On page 4 they discuss age at menarche as a risk factor for "cancer", in reality this is not an established risk factor for all cancers, only certain ones (mainly hormonal) and I'm not sure I would place this together with height. I suggest some rephrasing.

 2. on page 5 the authors not that case-control studies often suffer from information bias as the outcome has already been assessed. I think this sentence could be more clear to reflect the type of information bias they are implicating, and that it is not the outcome that has information bias (presumably) it is the exposure.

Is the topic of the opinion article discussed accurately in the context of the current literature?

Yes

Are arguments sufficiently supported by evidence from the published literature?

Yes

Are all factual statements correct and adequately supported by citations?

Yes

Are the conclusions drawn balanced and justified on the basis of the presented arguments?

Yes

Reviewer Expertise:

Epidemiology, Childhood Cancer

I confirm that I have read this submission and believe that I have an appropriate level of expertise to confirm that it is of an acceptable scientific standard.

# Response

**Published:** 2025-01-23

**Authors:** Vanessa Gordon-Dseagu

**Affiliations:** World Cancer Research Fund International, London, England, UK

The authors thank the reviewer for their kind words about our paper and for taking the time to carefully review it. We agree with both of their suggested changes.

**1. Reviewer query: **On page 4 they discuss age at menarche as a risk factor for "cancer", in reality this is not an established risk factor for all cancers, only certain ones (mainly hormonal) and I'm not sure I would place this together with height. I suggest some rephrasing.

**Author response:** We have amended the sentences related to age at menarche - although we have left it within the same paragraph as height because we believe the extra information adds clarity.

**2. Reviewer query: ** on page 5 the authors not that case-control studies often suffer from information bias as the outcome has already been assessed. I think this sentence could be more clear to reflect the type of information bias they are implicating, and that it is not the outcome that has information bias (presumably) it is the exposure.

**Author response:** We have added further information and detail to the section related to case-control studies and information bias - clarity has been given related to the bias relating to the exposure rather than the outcome only.
